# Henoch-Schonlein purpura associated with primary active Epstein-Barr virus infection: a case report

**DOI:** 10.11604/pamj.2017.27.29.10481

**Published:** 2017-05-11

**Authors:** Burcu Karakayali, Sila Yilmaz, Deniz Çakir, Pembe Gül Günes, Sirin Güven, Ismail Islek

**Affiliations:** 1 University of Health Sciences, Umraniye Research and Training Hospital, Department of Pediatrics, Istanbul, Turkey; 2 Haydarpasa Numune Research and Training Hospital, Department of Pathology, Istanbul, Turkey

**Keywords:** Henoch-Schönlein purpura, Epstein-Barr virus, children

## Abstract

Henoch-Schönlein purpura (HSP) is the most common form of childhood vasculitis. Various viral and bacterial infections, drugs, vaccines, food allergy and even insect bites have been considered as triggering factors in pathogenesis of HSP. Epstein-Barr virus (EBV) infection, which is associated with HSP, have been rarely reported. Herein we present HSP patient possibly caused by EBV infection. A 8-year old boy was admitted to our department with fever, rashes on legs and arms and intermittent mild abdominal pain. Multiple purpuric rashes were on his extremities, abdomen and buttock. Laboratory investigations revealed that monospot test was positive, EBV serology tests; Anti-EA-D Ig G: 3+, Anti-VCA gp125 Ig G: 3+, Anti-VCA p19 Ig M: 2+, Anti EBNA-1 Ig M: negative, Anti EBNA-1 Ig M: negative, Anti EBNA-1 Ig G: negative. The patient was interpreted as the primary active acute EBV infection. A skin biopsy showed leucocytoclastic vasculitis. The other viral and bacterial investigations were negative. The patient was diagnosed as HSP vasculitis according to EULAR criteria and treated with intravenous hydration and ibuprofen. He was discharged after 15 days with normal laboratory findings and physical examination. We think that EBV infection may be stimulant factor for autoimmune reactions and may cause HSP vasculitis. Hence, it may be useful to investigate the EBV infection in etiology of HSP cases.

## Introduction

Henoch-Schönlein purpura (HSP), which is the most common form of childhood vasculitis, affects mostly skin, joints, gastrointestinal system (GIS), kidneys, and in addition more rarely can have effect on central nervous system (CNS), heart and scrotum. Its pathogenesis and causal factors have not been accurately identified yet [1]. Viral and bacterial infections, drugs, vaccines, food allergy and even insect bites have been considered in the etiology of HSP. Streptococcus, vaccines, viral infections (varicella, measles, rubella, hepatitis A, B), tuberculosis, mycoplasma, Bartonella, helicobacter pylori are stated as the triggering factors in the literature [1–4]. Epstein-Barr virus (EBV) infection associated with HSP cases have been reported rarely [5–7]. Herewith we report the case of an 8-year old boy with primary active Epstein Barr Virus infection triggering HSP.

## Patient and observation

**Figure 1:**
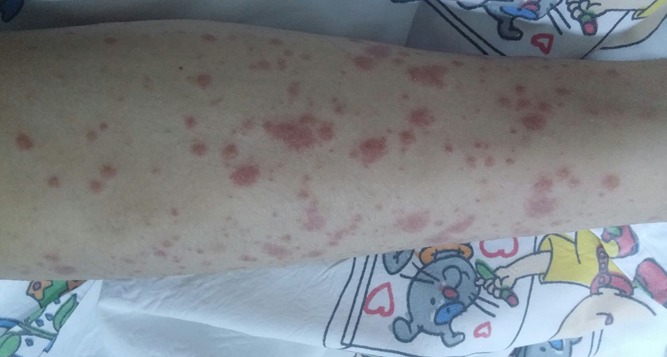
Multipl purpura on the lower extremity

**Figure 2:**
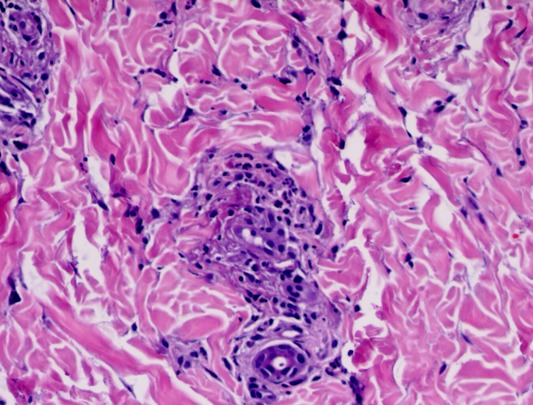
Vascular damage characterized by endothelial swelling and necrosis, fibrinoid change in vessel walls (HEX200)

A 8-year old boy, who was previously well, presented to the our emergency department with fever, fatigue, rashes on his both legs and arms and intermittent mild abdominal pain. On physical examination, the vital signs were as follows: blood pressure 110/60 mmHg, respiratory rate, 24/min; body temperature 38.5°C; heart rate, 110/min. Left submandibular lymph node was 2x1 cm, tender and soft. His lung auscultation and heart sounds were normal without murmur. His liver is palpable 1 cm below right costal margin. Splenomegaly was also found; confirmed by abdominal ultrasonography. Multiple purpuric rashes were on his extremities, abdomen and buttock Figure 1. On admission, laboratory investigations revealed white blood cell (WBC) count of 30,000/mm^3^, 75% lymphocytes, hemoglobin of 11 g/dl and a platelet count of 357,000/mm^3^. Biochemical studies revealed serum creatinine of 0.6 mg/dl, blood urea nitrogen of 19 mg/dl, protein 7.9 g/dl, serum albumin of 3.9 g/dl, aspartate amino transferase (AST) 181 IU/L (<52), alanine aminotransferase (ALT) 122 IU/L (<52) and gamma-glutamyltranspeptidase (GGT) 32 g/dL. Erythrocyte sedimentation rate and C-reactive protein level were 48 mm/h and 4,6 mg/dl(0-0,5 mg /dl) respectively. Blood coagulation tests, C3, C4, ANA, other immunoglobulin levels, anti-dsDNA, rheumatoid factor (RF), c-ANCA, p-ANCA and anti streptolysin-O (ASO) were negative. The investigation of Hepatitis B and C serologies, HIV, VDRL-RPR, VZV, CMV, toxoplasma, rubella Ig M and parvovirus antibody were all negative. Monospot test was positive. The EBV serology tests were revealed as follows Anti-EA-D Ig G: 3+, Anti-VCA gp125 Ig G: 3+, Anti-VCA p19 Ig M: 2+, Anti EBNA-1 Ig M: negative, Anti EBNA-1 Ig M: negative, Anti EBNA-1 Ig G: negative. In light of this findings, the patient was interpreted as the primary active acute EBV infection. In addition a skin biopsy from left malleol showed leucocytoclastic vasculitis Figure 2. Chest X-ray and an electrocardiogram were normal. The patient was diagnosed as HSP vasculitis according to criteria set by European League against Rheumatism (EULAR) [8]. The patient was treated with intravenous hydration and ibuprofen. The clinical finding improved gradually within 15 days.

## Discussion

We herein report the patient with HSP associated with primary active EBV infection. Our patient met clinically HSP criteria and leucocytoclastic vasculitis was demonstrated by skin biopsy. Viral and bacterial investigation showed primary active acute EBV infection and excluded the other infections. A variety of patients involving HSP and infection have been described following bacterial and viral infections [1–4]. EBV is also reported in association with various vasculitis forms such as Kawasaki disease, leucocytoclastic vasculitis, granulomatous vasculitis, systemic lupus erythematosus and ANCA associated vasculitis [9]. Pender stated autoimmune diseases are mainly caused by the infection of autoreactive B cells by EBV [10]. He suggested that autoimmune disease, as a result of EBV infection, is due interaction between B cells, already infected by EBV, and autoreactive T cells. The reason behind the damage of target organ in autoimmune disease is autoreactive T cells proliferation and stimulation to produce cytokines. In our patient, EBV antibodies results indicate primary active EBV infection [10]. Three cases, in which chronic EBV infection lead to a case similar to HSP, have been reported in the literature [4]. In addition, another three cases have been reported for acute EBV infection associated with HSP nephritis [6, 7]. Another case report presents a child patient who has acute EBV infection with immunecomplex glomerulonephritis resulting with hemodialysis need and resistant hypertension [5]. Three adult cases shows that EBV related lymph node lesions are similar to autoimmune disease related lymph node reactions [11].

## Conclusion

Summary, we think that EBV infection may be stimulant factor for autoimmune reactions and may cause HSP vasculitis. Hence, it may be usefull to investigate the EBV infection in etiology of HSP cases.

## Competing interests

The authors declare no competing interests.
